# Diagnostic value of circulating miR-21: An update meta-analysis in various cancers and validation in endometrial cancer

**DOI:** 10.18632/oncotarget.12028

**Published:** 2016-09-15

**Authors:** Yun Gao, Meiyu Dai, Haihua Liu, Wangjiao He, Shengzhang Lin, Tianzhu Yuan, Hong Chen, Shengming Dai

**Affiliations:** ^1^ Medical Science Laboratory, The Fourth Affiliated Hospital of Guangxi Medical University, Liuzhou, Guangxi 545005, China; ^2^ Department of Thoracic Surgery, The Fourth Affiliated Hospital of Guangxi Medical University, Liuzhou, Guangxi 545005, China; ^3^ Department of Haematology, The Fourth Affiliated Hospital of Guangxi Medical University, Liuzhou, Guangxi 545005, China

**Keywords:** miR-21, cancers, meta-analysis, diagnosis, endometrial carcinoma

## Abstract

MiR-21 has been identified as one of the most common proto-oncogenes. It is hypothesized that up-regulated miR-21 could be served as a potential biomarker for human cancer diagnosis. However, inconsistencies or discrepancies about diagnostic accuracy of circulating miR-21 still remain. In this sense, miR-21′s diagnostic value needs to be fully validated. In this study, we performed an update meta-analysis to estimate the diagnostic value of circulating miR-21 in various human cancers. Additionally, we conducted a validation test on 50 endometrial cancer patients, 50 benign lesion patients and 50 healthy controls. A systematical literature search for relevant articles was performed in Pubmed, Embase and Cochrane Library. A total of 48 studies from 39 articles, involving 3,568 cancer patients and 2,248 controls, were included in this meta-analysis. The overall sensitivity, specificity, positive likelihood ratio (PLR), negative likelihood ratio (NLR), diagnostic odds ratio (DOR) and area under the curve (AUC) were 0.76 (0.71-0.80), 0.82 (0.79-0.85), 4.3 (3.6-5.1), 0.29 (0.24-0.35), 15 (11-20) and 0.86 (0.83-0.89), respectively. In the validation test, the expression levels of serum miR-21 were significantly higher in benign lesion patients (*p* = 0.003) and endometrial cancer patients (*p* = 0.000) compared with that of healthy controls. Endometrial cancer patients showed higher miR-21 expression levels (*p* = 0.000) compared with benign lesion patients. In conclusion, the meta-analysis shows that circulating miR-21 has excellent performance on the diagnosis for various cancers and the validation test demonstrates that serum miR-21 could be served as a novel biomarker for endometrial carcinoma.

## INTRODUCTION

Early surveillance and diagnosis can potentially reduce the mortality of human cancers. Conventional diagnostic methods such as biopsy and imageological examination are invasive or harmful. Meanwhile, the diagnostic capability of common serum-based cancer biomarkers, such as carcinoembryonic antigen (CEA), alpha-fetalprotein (AFP) or carbohydrate antigen (CA), are also restricted due to the low sensitivity and specificity of these biomarkers. Consequently, a non-invasive and convenient diagnostic method with high sensitivity and specificity for human cancers is urgently needed.

MicroRNAs (miRNAs) are a class of small (19–25 nucleotides), non-coding and single-stranded RNAs that enhance or inhibit mRNAs expression at the post-transcriptional level [1, 2]. Over the past years, an increasing number of miRNAs, as oncogenes or tumor suppressor genes, have been proved differential expressions in a variety of cancers [3]. Studies also show that miRNAs are chemically stable and detectable in tissue, serum, plasma, urine, cerebrospinal fluid, feces as well as other body fluids [4]. The above findings demonstrate that circulating miRNAs could be used as novel biomarkers for human cancer screening and diagnosis for its superiorities of being non-invasive and convenient with high sensitivity and specificity [5].

MiR-21 has been identified as one of the most prominent oncogenic miRNAs and has been proved up-regulated in various human cancers [6, 7], which regulates the expression of multiple cancer-associated target genes [8–10]. Accumulating evidence strongly supports the role of miR-21 as oncogene in human cancers [11–19]. Therefore, it is hypothesized that up-regulated miR-21 could be employed as a potential biomarker for human cancer diagnosis. To date, numerous studies have testified the diagnostic value of circulating miR-21 in various human cancers [7, 20]. However, inconsistencies or heterogeneities about diagnostic accuracy of circulating miR-21 still remain and its diagnostic value in human cancers needs to be confirmed. In this study, we performed an update meta-analysis to assess the diagnostic value of circulating miR-21 in various human cancers.

As we all know, endometrial cancer has become one of dominant female cancers. But according to our meta-analysis, we only found one study focusing on the diagnostic value of circulating miR-21 in patients with endometrial cancer [21] and this study were excluded from our meta-analysis for the following reasons: (1) insufficient data to execute a two-by-two table; (2) only 12 cancer samples included which was less than 20. Given this, a validation test about serum miR-21 expression levels was also conducted among 50 endometrial cancer patients, 50 benign lesion patients and 50 healthy controls to make up the deficiency in diagnostic studies on circulating miR-21 of endometrial cancer.

## RESULTS

### Study selection and characteristics

**Figure 1:**
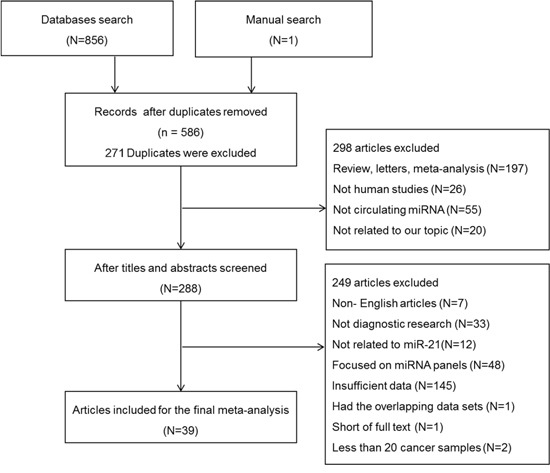
Flow diagram of study selection process based on inclusion and exclusion criteria

A total of 857 articles were identified by database search or manual search, among which 271 articles were excluded for duplicated data. After screening the titles and abstracts, 298 articles were excluded because they were review articles, letters, meta-analyses, non-human studies, studies on non-circulating miRNA or irrelevant to our topic. After reading and evaluating the full texts of the remaining 288 articles carefully, 249 articles were excluded in line with the exclusion criteria (7 articles were non-English articles; 33 articles were not diagnostic research; 12 articles were not related to miR-21; 48 articles focused on miRNA panels; 145 articles did not provide sufficient data; 1 article had the overlapping data sets; 1 article was short of full text; 2 articles collected less than 20 cancer samples). Finally, 48 studies from 39 articles were included in this meta-analysis [9, 22–59]. The flow diagram of study selection was shown in Figure 1.

On the whole, 48 studies from 39 articles published as of 3 March 2016, involving 3,568 cancer patients and 2,248 controls, were included in this meta-analysis. The sample types included serum (n = 27), plasma (n = 19) and peripheral blood mononuclear cell (PBMC) (n = 2). The cancer types included lung cancer (n = 10), colorectal cancer (n = 7), gastric cancer (n = 7), hepatocellular cancer (n = 6), breast cancer (n = 7), esophageal cancer (n = 2), pancreatic cancer (n = 1), head and neck squamous cell cancer (n = 1), nasopharyngeal cancer (n = 1), lymphoma (n = 1), biliary tract cancer (n = 2), laryngeal squamous cell cancer (n = 1), prostatecancer (n = 1) and retinoblastoma (n = 1). The miR-21 expression levels were detected through quantitative reverse transcription polymerase chain reaction (qRT-PCR) in all the studies, including 25 studies with Taqman probe and 23 studies with SYBR dye. The basic characteristics of the included articles were listed in Table 1.

**Table 1:** Characteristics and quality assessment of 48 studies included in meta-analysis

First author	Year	Country	Ethnicity	Patients (controls)	Cancer	Sample	method	ECon	Sen	Spe	Tp	Fp	Fn	Tn	AUC
Wei J[23]	2011	China	Asian	77(36)	LC	Plasma	SYBR	miR-16	61.04	83.33	47	6	30	30	0.729
Shen J[24]	2011	USA	Caucasian African	58(29)	LC	Plasma	SYBR	miR-16	79.31	65.52	46	10	12	19	0.816
Li Y[25]	2011	China	Asian	20(10)	LC	Serum	SYBR	mimics	78.80	100.00	16	0	4	10	0.912
Le HB[26]	2012	China	Asian	82(50)	LC	Serum	Taqman	miR-16	46.30	92.00	38	4	44	46	0.686
Wang B[9]	2012	China	Asian	31(39)	LC	Serum	SYBR	miR-16	87.10	74.40	27	10	4	29	0.880
Tang D[27]	2013	China	Asian	62(60)	LC	Plasma	Taqman	RNU6	48.40	78.30	30	13	32	47	0.715
Tang D[27]	2013	China	Asian	34(32)	LC	Plasma	Taqman	RNU6	52.90	71.90	18	9	16	23	0.709
Abd-EI-Fattah AA[28]	2013	Egypt	African	65(37)	LC	Serum	SYBR	RNU48	85.70	86.50	56	5	9	32	0.850
Mozzoni P[29]	2013	Italy	Caucasian	54(46)	LC	Plasma	Taqman	miR-16	50.00	92.30	27	4	27	42	0.740
Yang JS[56]	2014	China	Asian	300(152)	LC	Serum	Taqman	RNU6	69.00	71.00	207	45	93	107	0.810
Kanaan Z[57]	2012	USA	Caucasian	50(50)	CC	Plasma	Taqman	RNU6	90.00	90.00	45	5	5	45	0.820
Wang B[9]	2012	China	Asian	32(39)	CC	Serum	SYBR	miR-16	87.50	74.40	28	10	4	29	0.850
Toiyama Y[30]	2013	Japan	Asian	186(53)	CC	Serum	Taqman	cel-miR-39	82.80	90.60	154	5	32	48	0.919
Liu GH[31]	2013	China	Asian	200(80)	CC	Serum	Taqman	miR-16	65.00	85.00	130	12	70	68	0.802
Luo X[32]	2013	Germany	Caucasian	80(144)	CC	Plasma	Taqman	miR-16	51.70	80.70	41	28	39	116	0.653
Basati G[33]	2014	Iran	Caucasian	40(40)	CC	Serum	SYBR	RNU6	77.00	78.00	31	9	9	31	0.870
Ogata-kawata H[34]	2014	Japan	Asian	88(11)	CC	Serum	Taqman	miR-451	61.40	90.90	54	1	34	10	0.798
Tsujiura M[35]	2010	Japan	Asian	69(30)	GC	Plasma	Taqman	RNU6	60.90	63.33	42	11	27	19	0.673
Zheng Y[36]	2011	China	Asian	53(20)	GC	Plasma	SYBR	RNU6	83.77	80.53	44	4	9	16	0.853
Li BS[37]	2012	China	Asian	60(60)	GC	Plasma	Taqman	cel-miR-39	74.29	75.71	45	15	15	45	0.794
Wang B[9]	2012	China	Asian	30(39)	GC	Serum	SYBR	miR-16	56.70	94.90	17	2	13	37	0.810
Shiotani A[38]	2013	Japan	Asian	64(64)	GC	Serum	Taqman	miR-16	58.60	86.10	38	9	26	55	0.720
Wu J[22]	2015	China	Asian	50(50)	GC	Serum	SYBR	RNU6	83.77	79.60	44	10	6	40	0.912
Wu J[22]	2015	China	Asian	50(50)	GC	PBMC	SYBR	RNU6	74.29	73.40	41	13	9	37	0.898
Xu J[39]	2011	China	Asian	101(89)	HCC	Serum	SYBR	miR-181	56.70	73.50	85	24	16	65	0.870
Tomimaru Y[40]	2012	Japan	Asian	126(50)	HCC	Plasma	Taqman	miR-16	87.30	92.00	110	4	16	46	0.953
Tomimaru Y[40]	2012	Japan	Asian	126(30)	HCC	Plasma	Taqman	miR-16	61.10	83.30	77	5	49	25	0.773
Liu AM[41]	2012	China	Asian	57(59)	HCC	Serum	Taqman	NA	89.47	71.19	51	17	6	42	0.865
Amr KS[42]	2015	Egypt	African	23(17)	HCC	Serum	Taqman	RNU48	100.00	81.20	23	3	0	14	0.943
Zhuang C[59]	2015	China	Asian	52(43)	HCC	Serum	SYBR	cel-miR-39 RNU6	67.40	55.80	35	19	17	24	0.621
Asaga S[43]	2011	USA	Caucasian	79(20)	BC	Serum	SYBR	miR-16	67.00	75.00	53	5	26	15	0.721
Wang B[9]	2012	China	Asian	50(39)	BC	Serum	SYBR	miR-16	80.00	87.70	40	5	10	34	0.880
Mar-Aguilar F[44]	2013	Mexico	Caucasian	60(10)	BC	Serum	Taqman	18s RNA	94.40	80.00	57	2	3	8	0.950
Gao J[45]	2013	China	Asian	89(55)	BC	Serum	SYBR	miR-16	87.60	87.30	78	7	11	48	0.929
Toraih EA[46]	2015	Egypt	African	30(60)	BC	Serum	Taqman	RNU6	66.70	86.70	20	8	10	52	0.800
Motawi TM[58]	2016	Egypt	African	50(25)	BC	Serum	SYBR	RNU48	96.00	92.00	48	2	2	23	0.984
Motawi TM[58]	2016	Egypt	African	50(25)	BC	Serum	SYBR	RNU48	82.00	76.00	41	6	9	19	0.855
Kurashige J[47]	2012	Japan	Asian	71(39)	EC	Serum	Taqman	miR-16	46.50	100.00	33	0	38	39	NA
Wang B[9]	2012	China	Asian	31(39)	EC	Serum	SYBR	miR-16	71.00	69.20	22	12	9	27	0.740
Wang J[48]	2009	USA	Caucasian	49(36)	PC	Plasma	Taqman	miR-16	46.00	89.00	23	4	26	32	0.620
Hsu CM[49]	2012	China	Asian	50(36)	HNSCC	Plasma	Taqman	cel-miR-39	83.30	51.10	42	18	8	18	0.741
Liu X[50]	2013	China	Asian	217(73)	NPC	Plasma	SYBR	RNU6	76.00	69.90	165	22	52	51	0.792
Kishimoto T[51]	2013	Japan	Asian	94(50)	BTC	Plasma	Taqman	miR-16	85.10	100.00	80	0	14	50	0.930
Kishimoto T[51]	2013	Japan	Asian	94(23)	BTC	Plasma	Taqman	miR-16	72.30	91.30	68	2	26	21	0.830
Jones K[52]	2014	Australia	Caucasian	42(20)	Lym	Plasma	SYBR	cel-miR-39	95.00	86.00	40	3	2	17	0.920
Wang J[53]	2014	China	Asian	52(49)	LSCC	Serum	SYBR	RNU6	69.20	81.60	36	9	16	40	0.801
Huang W[54]	2015	China	Asian	75(75)	PCa	PBMC	Taqman	RNU6	87.50	85.70	66	11	9	64	0.833
Liu SS[55]	2014	China	Asian	65(65)	RB	Plasma	SYBR	RNU6	46.00	72.00	30	18	35	47	0.548

Sen: sensitivity, Spe: specificity, Econ: endogenous control, Tp: true positive, Fp: false positive, Fn: false negative, Tn: true negative, AUC: area under ROC curve, LC: lung cancer, CC: colorectal cancer, GC: gastric cancer, HHC: hepatocellular cancer, BC: breast cancer, EC: esophageal cancer, PC: pancreatic cancer, HNSCC: head and neck squamous cell cancer, NPC: nasopharyngeal cancer, BTC: biliary tract cancer, Lym: Lymphoma, LSCC: laryngeal squamous cell cancer, PCa: prostate cancer, RB: retinoblastoma, PBMC: peripheral blood mononuclear cell, NA: not available

The qualities of included articles were assessed by the Quality Assessment of Diagnostic Accuracy Studies (QUADAS) (Table 2) [60]. Item 1 was scored as “no” because samples with the known disease were recruited in each study. Item 2 was scored as “yes” because selection criteria were clearly described in each study. Item 3 was scored as “yes” because the reference standard could classify the target condition in each study. No studies stated the interval time between reference standard and index test so Item 4 was scored as “unclear”. Item 5 was scored as “yes” because reference standard was used in each study. Item 6 was scored as “yes” when all patients receive verification of the reference standard, or it would be scored as “unclear” when this information is not reported. Item 7 was scored as “yes” because the reference standard was independent of the index test in each study. For all the studies, the index test and the reference standard were described in detail so Item 8 and 9 were scored as “yes”. For Item 10, it was unclear if the index test was conducted without knowledge of the results from the reference standard. Item 11 was scored as “yes” because the reference standard results were defined without knowledge of the results from the index test. Item 12 was scored as “yes” because clinical data was available. Item 13 was scored as “yes” because uninterpretable test results were reported. Item 14 was scored as “yes” because withdrawals from the study were explained. With these references, the qualities of included articles were scored from 10 to 11 in this meta-analysis, which indicated that the quality of the included studies was satisfactory.

**Table 2:** QUADAS assessment for the studies included in meta-analysis for diagnosis

First author	Item 1	Item 2	Item 3	Item 4	Item 5	Item 6	Item 7	Item 8	Item 9	Item 10	Item 11	Item 12	Item 13	Item 14	Q
Wei J[23]	N	Y	Y	U	Y	Y	Y	Y	Y	U	Y	Y	Y	Y	11
Shen J[24]	N	Y	Y	U	Y	U	Y	Y	Y	U	Y	Y	Y	Y	10
Li Y[25]	N	Y	Y	U	Y	Y	Y	Y	Y	U	Y	Y	Y	Y	11
Le HB[26]	N	Y	Y	U	Y	Y	Y	Y	Y	U	Y	Y	Y	Y	11
Wang B[9]	N	Y	Y	U	Y	Y	Y	Y	Y	U	Y	Y	Y	Y	11
Tang D[27]	N	Y	Y	U	Y	Y	Y	Y	Y	U	Y	Y	Y	Y	11
Abd-EI-Fattah AA[28]	N	Y	Y	U	Y	Y	Y	Y	Y	U	Y	Y	Y	Y	11
Mozzoni P[29]	N	Y	Y	U	Y	Y	Y	Y	Y	U	Y	Y	Y	Y	11
Yang JS[56]	N	Y	Y	U	Y	Y	Y	Y	Y	U	Y	Y	Y	Y	11
Kanaan Z[57]	N	Y	Y	U	Y	Y	Y	Y	Y	U	Y	Y	Y	Y	11
Toiyama Y[30]	N	Y	Y	U	Y	U	Y	Y	Y	U	Y	Y	Y	Y	10
Liu GH[31]	N	Y	Y	U	Y	U	Y	Y	Y	U	Y	Y	Y	Y	10
Luo X[32]	N	Y	Y	U	Y	Y	Y	Y	Y	U	Y	Y	Y	Y	11
Basati G[33]	N	Y	Y	U	Y	Y	Y	Y	Y	U	Y	Y	Y	Y	11
Ogata-kawata H[34]	N	Y	Y	U	Y	U	Y	Y	Y	U	Y	Y	Y	Y	10
Tsujiura M[35]	N	Y	Y	U	Y	U	Y	Y	Y	U	Y	Y	Y	Y	10
Zheng Y[36]	N	Y	Y	U	Y	Y	Y	Y	Y	U	Y	Y	Y	Y	11
Li BS[37]	N	Y	Y	U	Y	Y	Y	Y	Y	U	Y	Y	Y	Y	11
Shiotani A[38]	N	Y	Y	U	Y	Y	Y	Y	Y	U	Y	Y	Y	Y	11
Wu J[22]	N	Y	Y	U	Y	Y	Y	Y	Y	U	Y	Y	Y	Y	11
Xu J[39]	N	Y	Y	U	Y	Y	Y	Y	Y	U	Y	Y	Y	Y	11
Tomimaru Y[40]	N	Y	Y	U	Y	Y	Y	Y	Y	U	Y	Y	Y	Y	11
Liu AM[41]	N	Y	Y	U	Y	U	Y	Y	Y	U	Y	Y	Y	Y	10
Amr KS[42]	N	Y	Y	U	Y	Y	Y	Y	Y	U	Y	Y	Y	Y	11
Asaga S[43]	N	Y	Y	U	Y	Y	Y	Y	Y	U	Y	Y	Y	Y	11
Mar-Aguilar F[44]	N	Y	Y	U	Y	U	Y	Y	Y	U	Y	Y	Y	Y	10
Gao J[45]	N	Y	Y	U	Y	Y	Y	Y	Y	U	Y	Y	Y	Y	11
Toraih EA[46]	N	Y	Y	U	Y	Y	Y	Y	Y	U	Y	Y	Y	Y	11
Kurashige J[47]	N	Y	Y	U	Y	Y	Y	Y	Y	U	Y	Y	Y	Y	11
Wang J[48]	N	Y	Y	U	Y	Y	Y	Y	Y	U	Y	Y	Y	Y	11
Hsu CM[49]	N	Y	Y	U	Y	Y	Y	Y	Y	U	Y	Y	Y	Y	11
Liu X[50]	N	Y	Y	U	Y	Y	Y	Y	Y	U	Y	Y	Y	Y	11
Kishimoto T[51]	N	Y	Y	U	Y	Y	Y	Y	Y	U	Y	Y	Y	Y	11
Jones K[52]	N	Y	Y	U	Y	Y	Y	Y	Y	U	Y	Y	Y	Y	11
Wang J[53]	N	Y	Y	U	Y	Y	Y	Y	Y	U	Y	Y	Y	Y	11
Huang W[54]	N	Y	Y	U	Y	Y	Y	Y	Y	U	Y	Y	Y	Y	11
Liu SS[55]	N	Y	Y	U	Y	U	Y	Y	Y	U	Y	Y	Y	Y	10
Zhuang C[59]	N	Y	Y	U	Y	Y	Y	Y	Y	U	Y	Y	Y	Y	11
Motawi TM[58]	N	Y	Y	U	Y	Y	Y	Y	Y	U	Y	Y	Y	Y	11

Item 1: Was the spectrum of patients representative of the patients? Item 2: Were selection criteria clearly described? Item 3: Is the reference standard likely to classify the target condition? Item 4: Is the time period between reference standard and index test short enough? Item 5: Did the whole sample use a reference standard of diagnosis? Item 6: Did patients receive the same reference standard regardless of the index test result? Item 7: Was the reference standard independent of the index test? Item 8: Was the index test described in sufficient detail? Item 9: Was the reference standard described in sufficient detail? Item 10: Were the index test results interpreted without knowledge of the results of the reference standard? Item 11: Were the reference standard results interpreted without knowledge of the results of the index test? Item 12: Were the same clinical data available when test results were interpreted as would be available when the test is used in practice? Item 13: Were uninterpretable/intermediate test results reported? Item 14: Were withdrawals from the study explained? [60] N: No, Y: Yes, N: unclear, Q: quadas

### Overall diagnostic accuracy and subgroup analyses

**Figure 2:**
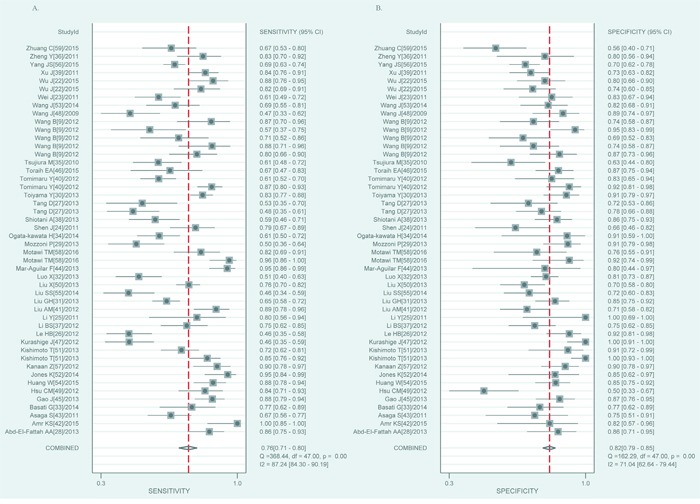
Forest plots for miR-21 in various cancers **A.** The pooled sensitivity. **B.** The pooled specificity.

**Figure 3:**
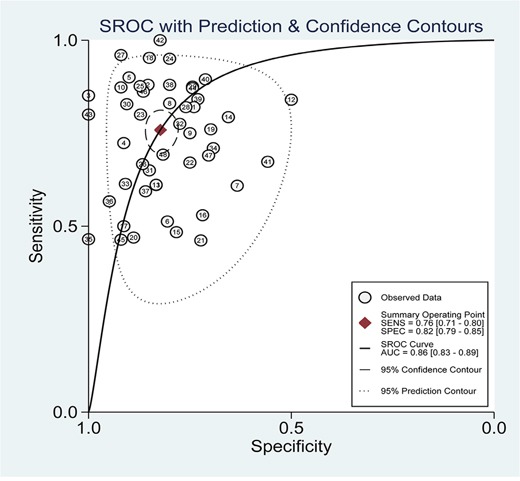
SROC curve of miR-21 for diagnostic value in various cancers

Heterogeneity across studies was determined and forest plots of sensitivity and specificity were shown in Figure 2. There appeared to be significant heterogeneity (for sensitivity, Q = 368.44, *p* = 0.00, I^2^ = 87.24; for specificity, Q = 162.29, *p* = 0.00, I^2^ = 71.04). Therefore, the random effects model was selected. The pooled results for diagnostic accuracy were shown in Table 3. The overall sensitivity, specificity, positive likelihood ratio (PLR), negative likelihood ratio (NLR), diagnostic odds ratio (DOR) and area under the curve (AUC) were 0.76 (0.71-0.80), 0.82 (0.79-0.85), 4.3 (3.6-5.1), 0.29 (0.24-0.35), 15 (11-20) and 0.86 (0.83-0.89), respectively. The summary receiver operator characteristic (SROC) curve was illustrated in Figure 3. The above results indicated that miR-21 had high diagnostic accuracy for various cancers.

**Table 3:** Summary results for diagnostic accuracy and their 95% confidence interval

Subgroup	Studies	Sensitivity (95%CI)	Specificity (95%CI)	PLR (95%CI)	NLR(95%CI)	DOR (95%CI)	AUC(95%CI)
Ethnicity							
Asian	34	0.74(0.68-0.78)	0.82(0.77-0.86)	4.1(3.2-5.2)	0.32(0.27-0.39)	13(9-18)	0.85(0.81-0.88)
Caucasian	8	0.77(0.59-0.89)	0.84(0.79-0.88)	4.8(3.3-6.8)	0.28(0.14-0.53)	17(7-44)	0.85(0.81-0.88)
African	5	0.89(0.75-0.96)	0.86(0.79-0.90)	6.2(4.0-9.5)	0.13(0.05-0.32)	49(15-154)	0.87(0.84-0.90)
Sample							
Serum	27	0.78(0.72-0.83)	0.83(0.78-0.86)	4.5(3.6-5.7)	0.26(0.21-0.34)	17(12-25)	0.88(0.84-0.90)
Plasma	19	0.71(0.63-0.78)	0.82(0.75-0.87)	3.9(2.8-5.5)	0.35(0.26-0.47)	11(6-20)	0.84(0.80-0.87)
Cancer							
LC	10	0.67(0.56-0.76)	0.81(0.74-0.86)	3.5(2.5-4.8)	0.41(0.31-0.55)	9(5-14)	0.82(0.79-0.85)
CC	7	0.75(0.63-0.83)	0.84(0.79-0.87)	4.6(3.4-6.3)	0.30(0.20-0.46)	15(8-30)	0.86(0.83-0.89)
GC	7	0.73(0.63-0.81)	0.80(0.73-0.86)	3.7(2.7-5.0)	0.33(0.24-0.46)	11(6-19)	0.84(0.80-0.87)
HCC	6	0.83(0.70-0.92)	0.77(0.66-0.86)	3.7(2.3-6.0)	0.21(0.11-0.42)	17(6-50)	0.87(0.83-0.89)
BC	7	0.85(0.75-0.91)	0.85(0.80-0.90)	5.8(4.0-8.5)	0.18(0.10-0.31)	33(14-76)	0.89(0.86-0.91)
Others	11	0.74(0.62-0.83)	0.86(0.74-0.93)	5.2(2.7-10.3)	0.31(0.21-0.45)	17(7-42)	0.86(0.83-0.89)
Method							
SYBR	23	0.80(0.74-0.84)	0.79(0.74-0.82)	3.7(3.0-4.6)	0.26(0.20-0.34)	14(9-22)	0.85(0.82-0.88)
Taqman	25	0.72(0.64-0.79)	0.85(0.80-0.89)	4.8(3.6-6.6)	0.33(0.25-0.42)	15(9-24)	0.87(0.84-0.90)
Endogenous control							
MiR-16	20	0.69(0.62-0.76)	0.87(0.82-0.91)	5.3(3.9-7.2)	0.35(0.28-0.44)	15(10-23)	0.87(0.83-0.89)
RNU6	14	0.74(0.65-0.81)	0.78(0.73-0.81)	3.3(2.6-4.2)	0.34(0.25-0.47)	10(6-17)	0.82(0.78-0.85)
cel-miR-39	4	0.84(0.676-0.89)	0.78(0.59-0.90)	3.8(1.9-7.7)	0.21(0.13-0.34)	18(6-52)	0.87(0.84-0.90)
Overall	48	0.76(0.71-0.80)	0.82(0.79-0.85)	4.3(3.6-5.1)	0.29(0.24-0.35)	15(11-20)	0.86(0.83-0.89)

CI: confidence interval, PLR: positive likelihood ratio, NLR: negative likelihood ratio, DOR: diagnostic odds ratio, AUC: area under ROC curve

In order to identify potential sources of heterogeneity, subgroup analyses were performed based on ethnic groups, sample types, cancer types, test methods and endogenous controls. Based on the results of subgroup analyses, the pooled sensitivity, specificity, PLR, DOR and AUC of Caucasian-based studies were 0.77 (0.59-0.89), 0.84 (0.79-0.88), 4.8 (3.3-6.8), and 17 (7-44) respectively, which were respectively higher than that of Asian-based studies (0.74 (0.68-0.78), 0.82 (0.77-0.86), 4.1 (3.2-5.2) and 13(9-18)). Subgroup analyses by sample types showed that the pooled sensitivity, specificity, PLR, NLR, DOR and AUC were 0.78 (0.72-0.83), 0.83 (0.78-0.86), 4.5 (3.6-5.7), 0.26 (0.21-0.34), 17 (12-25) and 0.88 (0.84-0.90) respectively for serum-based miR-21 assays, and 0.71 (0.63-0.78), 0.82 (0.75-0.87), 3.9 (2.8-5.5), 0.35 (0.26-0.47), 11 (6-20) and 0.84 (0.80-0.87) respectively for plasma-based miR-21 assays, showing that serum-based miR-21 assays had a higher performance than plasma-based miR-21 assays. The pooled sensitivity of SYBR-based studies was 0.80 (0.74-0.84), which was higher than that of Taqman-based studies (0.72 (0.64-0.79)). On the contrary, for Taqman-based studies, the pooled specificity, PLR, NLR, DOR and AUC (0.85 (0.80-0.89), 4.8 (3.6-6.6), 0.33 (0.25-0.42), 15 (9-24) and 0.87 (0.84-0.90)) were respectively higher than that for SYBR-based studies (0.79 (0.74-0.82), 3.7 (3.0-4.6), 0.26 (0.20-0.34), 14 (9-22) and 0.85 (0.82-0.88)). The pooled results for diagnostic accuracy were also shown in Table 3.

### Publication bias

**Figure 4:**
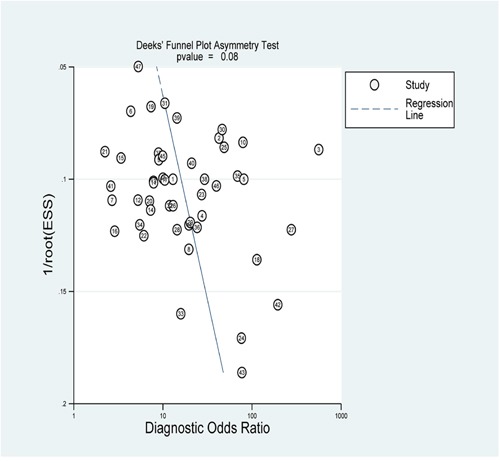
The Deek's test plot of the diagnostic meta-analysis

To assess the publication bias of the included studies, Deek's funnel plot was used in the meta-analysis. For our meta-analysis, the funnel plot was symmetric and the *p* value were 0.08 (Figure 4), indicating no significant publication bias occurred.

### Validation test on endometrial cancer patients

**Figure 5:**
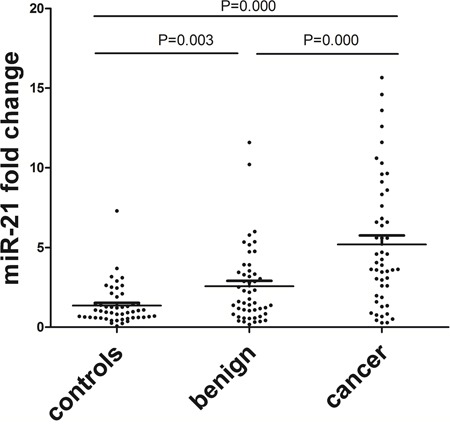
Relative fold change of serum miR-21 in endometrial cancer patients (n = 50), benign lesion patients (n = 50) and healthy controls (n = 50) Benign lesion patients vs. healthy controls, *p* = 0.003; endometrial cancer patients vs. healthy controls, *p* = 0.000 and benign lesion patients vs. endometrial cancer patients, *p* = 0.000.

**Figure 6:**
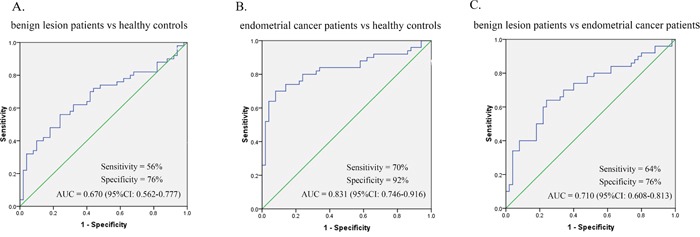
ROC curve analysis for evaluating serum miR-21 diagnostic performance **A.** The performance in differentiating benign lesion patients from healthy controls. **B.** The performance in differentiating endometrial cancer patients from healthy controls. **C.** The performance in differentiating benign lesion patients from endometrial cancer patients.

A total of 150 serum samples were detected, including those from endometrial cancer patients (n = 50), benign lesion patients (n = 50) and healthy controls (n = 50). No statistical differences in age were observed between healthy controls and benign lesion patients (*p* = 0.967), healthy controls and endometrial cancer patients (*p* = 0.992) or benign lesion patients and endometrial cancer patients (*p* = 0.983). The expression levels of serum miR-21 were significantly higher in benign lesion patients (*p* = 0.003) and endometrial cancer patients (*p* = 0.000) than that in healthy controls. Endometrial cancer patients showed higher miR-21 expression level (*p* = 0.000) compared with benign lesion patients (Figure 5). Receiver operating characteristic (ROC) curve was employed to assess the diagnostic value of serum miR-21 in benign lesion patients and endometrial cancer patients. Serum miR-21 showed an AUC value of 0.670 (0.562-0.777) in discriminating benign lesion patients from healthy controls. With a cutoff value of 1.502, the sensitivity and specificity were 56% and 76% respectively (Figure 6A). Serum miR-21 showed an AUC value of 0.831 (0.746-0.916) in discriminating endometrial cancer patients from healthy controls. With a cutoff value of 2.937, the sensitivity and specificity were 70% and 92% respectively (Figure 6B). Serum miR-21 showed an AUC value of 0.710 (0.608-0.813) in discriminating endometrial cancer patients from benign lesion patients. With a cutoff value of 3.457, the sensitivity and specificity were 64% and 76% respectively (Figure 6C).

## DISCUSSION

In this meta-analysis, a total of 48 studies from 39 articles were included, involving 3,568 cancer patients and 2,248 controls. The overall sensitivity and specificity for identifying various cancers were 0.76 and 0.82 respectively, which were much higher than that of traditional serum-based cancer biomarkers. In this meta-analysis, the overall PLR was 4.3, which suggests that the possibility of developing cancer for potential patients is 4.3 times higher than that of healthy controls when circulating miR-21 levels are elevated; the overall NLR was 0.29, which indicates that the possibility of developing cancer is 29% when circulating miR-21 assay is normal. Moreover, in order to evaluate the diagnostic performance of circulating miR-21 in human cancers, SROC curve was plotted and the AUC was 0.86, suggesting the diagnosis performance of circulating miR-21 was excellent. All in all, the above results indicated that circulating miR-21 had high diagnostic accuracy for various human cancers. In order to trace the sources of heterogeneity, subgroup analyses were performed based on ethnic groups, specimen types, cancer types, test methods and endogenous controls which might have potential to cause variable results.

Based on the results of subgroup analyses, the diagnostic accuracy of Caucasian-based studies was higher than that of Asian-based studies. As for the selection of blood types, collection methods can significantly influence the miRNAs concentration. In this meta-analysis, serum samples have better performance than plasma which suggests that serum may be suitable in the detection of circulating miR-21. SYBR and Taqman probe are the most frequently used methods of real-time quantitative PCR. To compare the characteristic of these two methods, the sensitivity of SYBR was higher than that of Taqman probe while the specificity showed reverse result. The choice of endogenous controls is a contentious issue in the detection of circulating miRNAs. At present, miR-16, RNU6 and caenorhabditis elegans miRNA (cel-miR-39) are the most commonly used endogenous controls for circulating miR-21. In this meta-analysis, the performance of these three endogenous controls appeared to be equally matched.

Although some meta-analyses related to diagnostic value of circulating miR-21 in human cancers have been previously reported, there are some following advantages in this meta-analysis: (I) 14 kinds of cancers are included in this meta-analysis; (II) compared with previous reported meta-analysis, much more studies and samples are included; (III) more comprehensive subgroup analyses than any other reported meta-analysis are conducted, including ethnic groups, sample types, cancer types, test methods and endogenous controls; (IV) a relatively systematic analysis about pre-analysis variables is performed in this meta-analysis. In conclusion, this study is the first systematic review and comprehensive diagnostic meta-analysis to evaluate the overall diagnostic accuracy of circulating miR-21 for various cancers.

There are still several limitations in this meta-analysis despite the advantages mentioned above. First, there are only eight Caucasian-based studies and five African-based studies, which may lead to statistical insufficiency. Second, the heterogeneity exists in this meta-analysis. Subgroup analyses are applied, but the results could not fully explain the observed heterogeneity.

Except for the meta-analysis, we also identified the diagnostic value of circulating miR-21 in patients with endometrial cancer or benign lesion in this study, which can make up the deficiency in diagnostic studies on circulating miR-21 of endometrial cancer. Compared with the previous study [21], our validation test is more convincing. First, our validation test could provide more comprehensive data for the diagnosis research, such as cutoff value, the sensitivity and the specificity. Second, our validation test has larger sample size. Third, our validation test is the first study to detect serum miR-21 expression levels of benign lesion patients. In our validation test, serum miR-21 showed excellent performance on the diagnosis of endometrial cancer (*p* = 0.000), which was consistent with the results of our meta-analysis. The ROC curve analysis revealed robust levels of serum miR-21 in discriminating endometrial cancer patients from control subjects (sensitivity = 70%, specificity = 92%, AUC = 0.831). We also observed significance by comparing data collected from benign lesion patients with that of healthy controls (*p* = 0.003) or endometrial cancer patients (*p* = 0.000). In conclusion, serum miR-21 expression levels were statistically up-regulated in patients with benign lesion and endometrial cancer. In other words, the serum miR-21 expression levels of benign lesion patients were higher than that of healthy controls but lower than that of endometrial cancer patients.

This study assessed the role of circulating miR-21 as a biomarker for the diagnosis of various cancers by meta-analysis and further validated the role of circulating miR-21 in endometrial cancer. In conclusion, the meta-analysis shows that circulating miR-21 has excellent performance on the diagnosis for various cancers and the validation test demonstrates that serum miR-21 could be served as a novel biomarker for endometrial carcinoma.

## MATERIALS AND METHODS

### Search strategy

A systematical literature search for relevant articles was performed in PubMed, Embase and Cochrane Library as of 3 March, 2016. Our search was performed based on the following key terms: (miR-21 or microRNA-21 or miRNA-21) and (cancer or tumor or carcinoma or malignancy or neoplasm) and (circulating or serum or sera or plasma or blood) and (diagnostic or diagnosis or sensitivity or specificity). In addition, the reference lists of related review articles were scanned for further screening.

### Inclusion and exclusion criteria

All articles included must be in accordance with the following criteria: (I) researches on patients with any type of human cancers; (II) cancers diagnosed by golden standard; (III) the relationship investigated between circulating miR-21 expression levels and cancer diagnosis; (IV) patients with benign diseases or healthy individuals as the control group; (V) sufficient data provided to execute a two-by-two table. Exclusion criteria were as follows: (I) review article, letter or meta-analysis; (II) non-English; (III) irrelevant to our topic; (IV) lack of key information; (V) less than 20 cancer samples. When the same patient groups were enrolled from more than one article, the largest and most comprehensive one was selected in this meta-analysis to avoid overlapping.

### Data extraction and quality assessment

The whole process of this meta-analysis was executed and assessed by two independent reviewers (Yun Gao and Meiyu Dai). When inconsistencies occurred, consensuses were achieved through detailed discussions. The following data were extracted for each eligible study: the first author, year of publication, country, ethnicity, sample size, sample type, cancer type, detecting method, endogenous control and diagnostic results including sensitivity, specificity, true positive, false positive, false negative, true negative and AUC. The QUADAS [60] was used to systematically evaluate the quality of included articles. The QUADAS checklist includes 14 items which are listed in the notes of Table 2. Each item is assessed by “yes”, “no” or “unclear”. The answer “yes” scores a point, whereas “no” or “unclear” scores zero.

### Validation test on endometrial cancer patients

In this study, we recruited 50 endometrial cancer patients, 50 benign lesion patients (uterine myoma and endometrial polyp) and 50 healthy controls matched in sex and age from the Fourth Affiliated Hospital of Guangxi Medical University. Endometrial cancer patients were confirmed by pathological diagnosis and the serum samples were collected before any treatment. Patients who had received chemotherapy, radiotherapy or operation were excluded from this study. This study was approved by institutional review board of the Fourth Affiliated Hospital of Guangxi Medical University.

MiRNA was extracted from 200μl serum using the miRNeasy Serum/Plasma Kit (Qiagen, CA, USA) according to the manufacturer's instructions. Cel-miR-39 was used as the reference gene. The reverse transcription reaction and real-time quantitative PCR were performed using miScript II RT Kit (Qiagen, CA, USA) and miScript SYBR Green PCR Kit (Qiagen, CA, USA) according to the manufacturer's instructions. The relative expression levels of miR-21 were calculated using 2^−ΔΔCt^ method.

### Statistical analysis

Meta-analysis was performed using the Stata 12.0 (Stata Corporation, College Station, TX, USA). The meta-analysis models were used to calculate pooled sensitivity, pooled specificity, PLR, NLR and DOR. The SROC curve was plotted and AUC was calculated to evaluate the performance of diagnostic studies. Heterogeneity was evaluated by the Q test and I^2^. When homogeneity is achieved (*p* ≥ 0.10, I^2^ ≤ 50%), a fixed-effect model is used for secondary analysis. If not (*p* < 0.10, I^2^ > 50%), a random-effect model is used to perform subgroup analyses for exploring sources of heterogeneity. Deek's funnel plot was performed to evaluate the publication bias. If *p* < 0.10, publication bias exists. In the validation test, statistical analyses were performed with SPSS 16.0 (SPSS, Inc., Chicago, IL). Statistical differences of miR-21 relative expression levels were evaluated by Mann-Whitney test. All tests were two sided and *p* < 0.05 was considered statistically significant. The ROC curve and AUC were performed to analyze the diagnostic value of miR-21. The cutoff value was obtained by Youden index.
